# The effect of measurement area size on the reliability of myocardial iron load measurement in cardiac magnetic resonance imaging examinations of thalassemia patients

**DOI:** 10.3906/sag-1804-139

**Published:** 2021-04-30

**Authors:** İbrahim Önder YENİÇERİ, Fatih Mehmet AZIK, Funda Dinç ELİBOL

**Affiliations:** 1 Department of Radiology, School of Medicine, Muğla Sıtkı Koçman University, Muğla Turkey; 2 Department of Pediatry, School of Medicine, Muğla Sıtkı Koçman University, Muğla Turkey

**Keywords:** Thalassemia major, myocardial ironload, T2* MRI, ROI size

## Abstract

**Background/aim:**

The aim of this study was to evaluate the intraobserver and interobserver reliability of cardiac T2* MRI measurements in different region of interest (ROI) sizes.

**Materials and methods:**

Cardiac T2* MRIs of 24 thalassemia major patients were evaluated. Two different ROI sizes were used for measurement. In the first measurement, an ROI approximately 5 mm in diameter was used in the interventricular septal myocardium. In the other method, the whole ventricular septal myocardium was used as the measurement. The intraobserver and interobserver variabilities were assessed with the intraclass correlation coefficient (ICC).

**Results:**

The measurement of the first observer, the ICC of the small-sized ROI (ssROI), was 0.869, and the measurement for the second observer, the ICC of the ssROI, was 0.659. The ICC of the whole-septal ROI (wsROI) was 0.991 for the first observer and 0.980 for the second observer. Interobserver variability, for the mean measurement, was 0.442 for the ICC of ssROI and 0.883 for the ICC of wsROI.

**Conclusion:**

For the evaluation of myocardial iron load with T2* MRI we suggest making measurements with ROI, including all of the interventricular septum, as a consequence of high intraobserver and interobserver consistency.

## 1. Introduction

In refractory anemias such as thalassemia major, there is iron overload in the internal organs, particularly the liver, heart, and pancreas, due to recurrent transfusions. In thalassemia major patients the heart is the most important organ because of its effect on mortality and morbidity [1]. There is a strong correlation between the serum ferritin level and iron load in internal organs [2]. Moreover, the serum ferritin level can be affected by inflammatory conditions [3]. Nonetheless, there is not a strong correlation between the serum ferritin level and cardiac iron load [4]. It is very important that the myocardial iron load be accurately diagnosed so that effective treatment can be administered. 

Today, noninvasive measurement of myocardial iron load is performed using T2* MRI and the software supplied by equipment manufacturers. There is a great amount of software available for this purpose. Despite technological improvements in MRI, due to the heterogeneity of myocardial iron distribution, MRI sequence standardization problems, and software problems, many clinicians are concerned about the evaluation of myocardial iron load with MRI [5]. Of particular note is the lack of consistency among repeated measurements (reliability), which makes accurate and confident diagnosis difficult. In addition, the literature lacks sufficient data concerning how region of interest (ROI) size affects measurement of myocardial iron load. The present study aimed to determine the intraobserver and interobserver reliability of several ROI sizes used for measurement of myocardial iron load via T2* MRI in thalassemia major patients.

## 2. Material and methods

### 2.1. Patients

The T2* MRI images of 24 thalassemia major patients (14 female and 10 male) obtained between June and September 2016 were retrospectively evaluated. Exclusion criteria included motion artifacts and artifacts due to the MRI technique. The study protocol was approved by the university ethics committee.

### 2.2. MRI

MRI was performed using a 1.5 T MR scanner (GE, Signa, USA). A T2*-weighted MRI was used to measure myocardial iron load using a fast gradient-echo multiecho sequence (8 echoes) and ECG triggering. Short-axis views (midventricular) of the left ventricle were obtained. Multiecho sequence parameters were as follows; matrix: 192 × 256 mm; flip angle: 25°; TR: 120 ms; TE: 1.5–13.31 ms; FOV: 41 × 41 mm; slice thickness: 5 mm.

### 2.3. Image analysis

Measurement was performed using CMRtools software (CMRtools Thalassemia-Tools, Cardiovascular Imaging Solutions, London, UK). The DICOM images of thalassemia patients in our hospital radiology archive were copied to local memory, and measurements were performed in the local memory. All T2* measurements were performed by 2 observers using midventricular short axis slices. In the first slice the ROI was drawn in the septum, and the ROI in the other 7 slices was automatically selected by CMRtools; however, it was possible to manually select the ROI in the latter 7 slices if necessary due to motion artifacts. 

**Figure 1:**
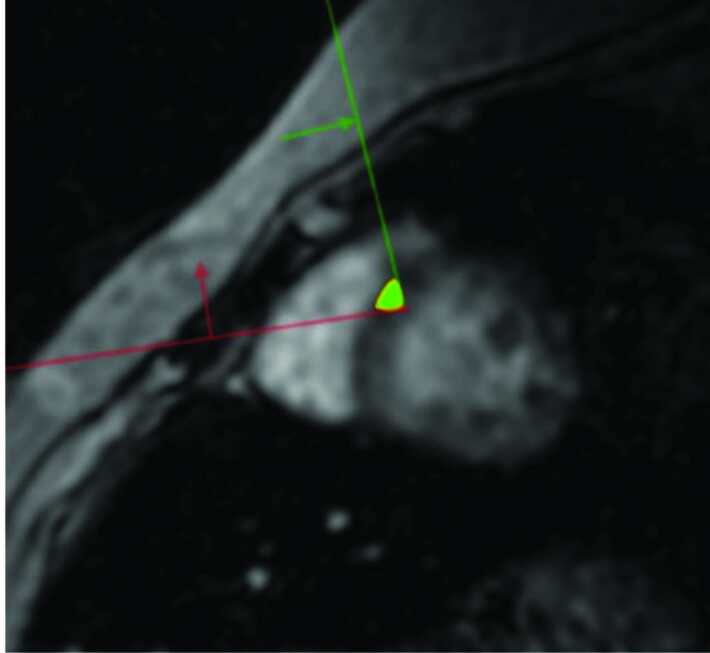
An ROI approximately 5 mm in diameter (ssROI) was used.

**Figure 2:**
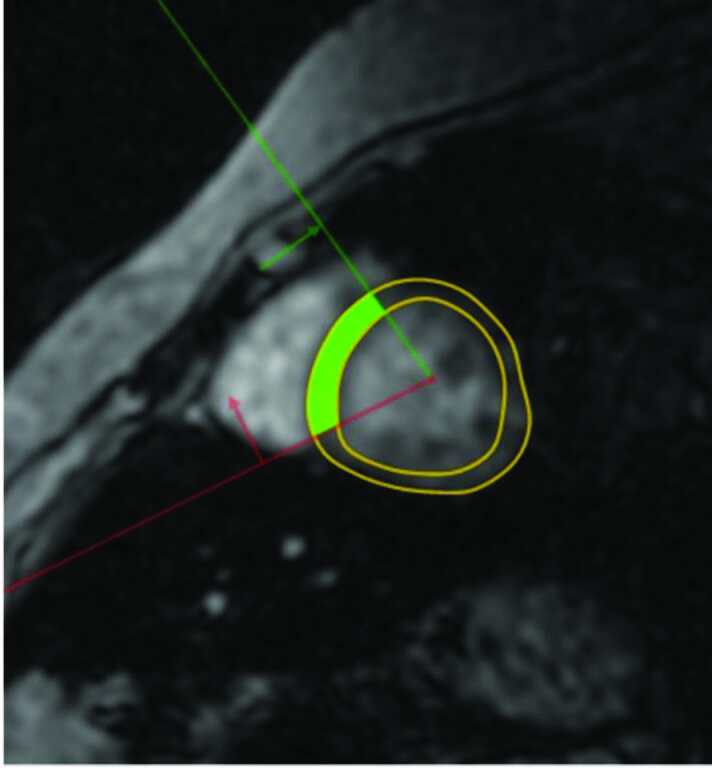
ROI including the entire septum (wsROI).

For measurement of the first slice, a ROI approximately 5 mm in diameter was used, and it was referred to as a small-sized ROI (ssROI). The lower limit for interventricular septum thickness is 6 mm; therefore, a 5-mm ROI was used to ensure that it included the entire septum, but none of the lumen. In fact, there are some centers that measure cardiac iron load by this type of method. The ssROI was drawn so that it did not include anything beyond the myocardium (Figure 1). The ssROI was selected manually by inputting 3 points in CMRtools. In the second measurement method, an inner and outer contour was drawn manually. Next, a reference ROI—including the septum—was defined automatically by CMRtools. The operator could make changes and arrange the area of ROI. This measurement was referred to as whole-septal ROI (wsROI) (Figure 2). The drawing did not extend beyond the myocardium because the signal of the blood can affect measurement. The ssROI and wsROI measurements were performed by 2 observers, with a 1-week interval between each measurement and blinding of the previous values and each other. The first observer had 1 year of experience with cardiovascular imaging and had been using CMRtools for 6 months. The second observer was performing T2* MRI measurements and using CMRtools for the first time. 

### 2.4. Statistical analysis

Intraobserver and interobserver reliability were assessed using the intraclass correlation coefficient (ICC). The ICC of ssROI and wsROI was calculated for both observers. For interobserver reliability, ICC estimates and their 95% confident intervals were calculated using SPSS version 20 (IBM Corp., Armonk, NY, USA) based on a mean-rating (k = 2), consistency-agreement, 2-way random-effects model. For intraobserver reliability, ICC estimates and their 95% confident intervals were calculated using SPSS version 20 based on a mean-measurement (k = 2), absolute-agreement, 2-way mixed-effects model. The ICC values less than 0.5 are indicative of poor reliability, values between 0.5 and 0.75 indicate moderate reliability, values between 0.75 and 0.9 indicate good reliability, and values greater than 0.90 indicate excellent reliability [6].

## 3. Results

Among the 24 patients, 14 (58.3%) were female and 10 (41.7%) were male. The mean age of the entire patient population was 26.08 years (range: 17–44 years), versus 26.27 years (range: 18–44 years) for the females and 25.67 years (range: 17–42 years) for the males. Observer measurements are summarized in Table 1. 

**Table 1:** Both observers made two measurements by two different methods. As a result, each observer made four measurements. All measurements of the observers are given in the table.

	Observer 1 measurement				Observer 2 measurement			
	wsROI-1	wsROI-2	ssROI-1	ssROI-2	wsROI-1	wsROI-2	ssROI-1	ssROI-2
N	24	24	24	24	24	24	24	24
Mean	39.6129	39.8896	31.2450	53.6283	40.3092	40.6679	30.3058	40.1025
Minimum	11.91	11.79	9.22	11.97	9.34	8.03	4.85	6.66
Maximum	117.15	106.00	141.57	208.55	109.75	108.74	55.94	166.02

ws: whole-septal, ss: small-sized, ROI: region of interest, N: number of cases.

For the first observer, the ICC of ssROI was 0.869 (0.697–0.943 in the 95% confidence interval), and the ICC of wsROI was 0.991 (0.978–0.996 in the 95% confidence interval). Among the measurements of observer 1, the reliability of the ssROI method was good, and the wsROI method was excellent (Table 2). For the second observer, the ICC of ssROI was 0.659 (0.211–0.852 in the 95% confidence interval), and the ICC of wsROI was 0.980 (0.954–0.991 in the 95% confidence interval). Among the measurements of observer 1, the reliability of the ssROI method was moderate, and the wsROI method was excellent (Table 3).

**Table 2:** Comparison of ssROI and wsROI measurements for observer 1.

			95% confidence interval		
		ICC coefficient	Lower bound	Upper bound	
ssROI	Avarage measure	0.869	0.697	0.943	P < 0.01
wsROI	Avarage measure	0.991	0.978	0.996	P < 0.01

**Table 3:** Comparison of ssROI and wsROI measurements for observer 2.

			95% confidence interval		
		ICC coefficient	Lower bound	Upper bound	
ssROI	Avarage measure	0.659	0.211	0.852	P < 0.01
wsROI	Avarage measure	0.980	0.954	0.991	P < 0.01

In terms of interobserver reliability, the ICC of ssROI was 0.442 (0.056–0.713 in the 95% confidence interval), and the ICC of wsROI was 0.883 (0.749–0.948 in the 95% confidence interval). In terms of observer measurements, the reliability of the ssROI method was poor, and the wsROI method was good–excellent (Table 4).

**Table 4:** Interobserver reliability of ssROI and wsROI measurements, based on both observers’ means.

			95% confidence interval		
		ICC coefficient	Lower bound	Upper bound	
ssROI	Avarage measure	0.442	0.056	0.713	P = 0.01
wsROI	Avarage measure	0.883	0.749	0.948	P < 0.01

**Figure 3:**
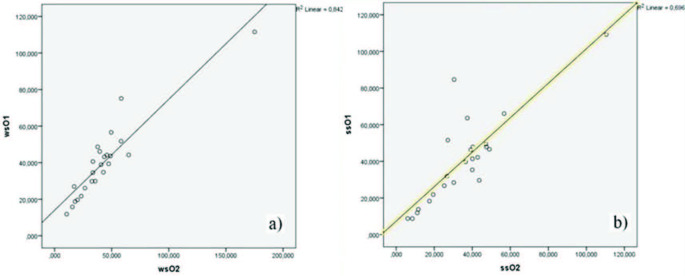
Interobserver reliability of measurements using the wsROI method (on the left) is slightly higher than that of measurements using the ssROI method (on the right). Comparisons were made using mean measurements.

**Figure 4:**
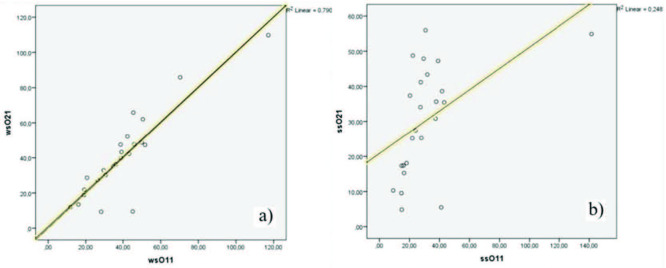
Interobserver reliability of measurements using the wsROI method (on the left) is slightly higher than that of measurements using the ssROI method (on the right). Comparisons were made using the first measurements of each observer.

Figure 3a and Figure 3b show that the interobserver measurement consistency of the wsROI method (R² = 0.842) was slightly higher than in the ssROI method (R² = 0.696). Because the values are the mean of both measurement methods, the R² values are close to each other. When we evaluated single measurements alongside mean values of T2* measurements, the R² values for each measurement method (wsROI and ssROI) were significantly different (Figures 4a and 4b). The first wsROI measurement value for each observer (R² = 0.790) approached the mean value; however, the first ssROI measurement for each observer was slightly lower (R² = 0.248). While single measurements using the wsROI method are as reliable as the mean of multiple measurements with the ssROI method, mean values obtained with the ssROI method in different parts of the septum show relatively reliable and reproducible results. 

## 4. Discussion

In the present study the ICC of wsROI was significantly higher than the ICC of ssROI. The wide variation in ssROI measurements might have been due to the heterogeneity of the myocardium iron load or MRI artifacts. In a study that included 14 idiopathic hemochromatosis patients, Olson et al. [7] emphasized heterogeneity of myocardial iron load in cardiac autopsies. They also suggested that iron load might be focal and recommended repeating endomyocardial biopsies. Presently, endomyocardial biopsies are not routinely used in the management of iron overload as they are invasive, and noninvasive modalities such as T2* MRI are readily available. 

A study of the heterogeneity of iron load in the myocardium reported a heterogenous iron load in 50% of thalassemia patients [8]. The researchers made multiple measurements in the myocardium using the multislice technique and obtained global T2*-weighted images of the myocardium. They showed that global myocardial T2* measurements correlated well with one measurement of the midventricular septum. Of note, the researchers used a whole septal ROI, which is important because a ROI of that size has the advantage of providing the mean of different parts of the myocardium. 

Roghi et al. observed significantly different myocardial T2-weighted values based on different tools [5]. They used CMRtools and the pixel-wise (PW) inline myocardial T2*-weighted mapping system for characterizing myocardial iron load in thalassemia major patients. They concluded that different methods can categorize iron accumulation differently, and that consecutive measurements of a patient may result in misinterpretation, especially of borderline values, which can negatively affect iron chelation treatment. Earlier studies have shown that there is a lack of standardization across iron load measurement methods, including the unit of measure [2,9]. In addition, as different measurement programs may categorize a patient differently, it is of paramount importance to standardize software in order to minimize intraobserver and interobserver variability.

The present study aimed to determine the effect of ROI size on the reliability of myocardial iron load measurements. Both intraobserver and interobserver ICC were higher for measurements obtained via wsROI than via ssROI, indicating that as ROI size decreased, measurement inconsistency increased. Figure 5 shows the measurements of both observers for one patient; the interobserver evaluation showed that the difference in measurements with the ssROI method was 19.28%, versus 1.12% with the wsROI method. Additionally, some software was measured with ssROI and using ssROI aroused suspicion regarding the repeatability of measurements. The main dilemma in this situation is that measurements should be used to establish management. In terms of chelation therapy, clinicians often must decide if they should use the highest iron load measurement or the mean load measurement of the septum; however, based on the results of long-term studies the mean overload measurement of the septum is the best choice. 

The present study has some limitations, including its retrospective design and the exclusion of a large number of patients due to MRI artifacts (primarily due to technician inexperience). To the best of our knowledge the present study is the first to examine the effect of ROI size on the reliability of T2* MRI measurement of myocardial iron load in thalassemia patients. In cardiac T2*-weighted measurements, some of the updated software was user-dependent, while some contained templates regarding ROI size and shape and measurements. Based on the present findings, we strongly advocate for standardization of T2* MRI measurement of myocardial iron load. 

## 5. Conclusion

Different measurements from consecutive T2* MRIs using ssROI in the same patient makes the monitoring, management, and treatment of thalassemia patients challenging. Based on the present findings, for the evaluation of myocardial iron load based on T2*-weighted MRI we suggest using a ROI that includes the entire septum, as it is associated with high intraobserver and interobserver reliability. Lastly, the present findings clearly show that as ROI size increases, the reliability of T2* MRI measurement of myocardial iron load increases.
